# A longitudinal analysis of PM_2.5_ exposure and multimorbidity clusters and accumulation among adults aged 45-85 in China

**DOI:** 10.1371/journal.pgph.0000520

**Published:** 2022-06-29

**Authors:** Kai Hu, Katherine Keenan, Jo Mhairi Hale, Yang Liu, Hill Kulu

**Affiliations:** 1 Population and Health Research Group, School of Geography and Sustainable Development, University of St Andrews, Fife, United Kingdom; 2 Gangarosa Department of Environmental Health, Rollins School of Public Health, Emory University, Atlanta, Georgia, United States of America; Kathmandu Medical College and Teaching Hospital: Kathmandu Medical College, NEPAL

## Abstract

While previous studies have emphasised the role of individual factors in understanding multimorbidity disparities, few have investigated contextual factors such as air pollution (AP). We first use cross-sectional latent class analysis (LCA) to assess the associations between PM_2.5_ exposure and multimorbidity disease clusters, and then estimate the associations between PM_2.5_ exposure and the development of multimorbidity longitudinally using growth curve modelling (GCM) among adults aged 45–85 in China. The results of LCA modelling suggest four latent classes representing three multimorbidity patterns (respiratory, musculoskeletal, cardio-metabolic) and one healthy pattern. The analysis shows that a 1 *μg*/m^3^ increase in cumulative exposure to PM_2.5_ is associated with a higher likelihood of belonging to respiratory, musculoskeletal or cardio-metabolic clusters: 2.4% (95% CI: 1.02, 1.03), 1.5% (95% CI: 1.01, 1.02) and 3.3% (95% CI: 1.03, 1.04), respectively. The GCM models show that there is a u-shaped association between PM_2.5_ exposure and multimorbidity, indicating that both lower and higher PM_2.5_ exposure is associated with increased multimorbidity levels. Higher multimorbidity in areas of low AP is explained by clustering of musculoskeletal diseases, whereas higher AP is associated with cardio-metabolic disease clusters. The study shows how multimorbidity clusters vary contextually and that PM_2.5_ exposure is more detrimental to health among older adults.

## Introduction

The number of older adults living with multimorbidity, defined as the coexistence of two or more chronic diseases or conditions, is rising globally [1]. Research based on survey data shows a high prevalence of multimorbidity among older adults in low and middle income countries (LMICs), such as 63% in India [2], 65% in Brazil [3], 69% in South Africa [4], and 46% in China [5]. The increasing prevalence of multimorbidity is associated with worse functional ability, reduced healthy life expectancy, increased mortality, and a higher rate of hospitalisations [6–8], leading to a heavy burden on medical and health systems and inequalities in health outcomes [9]. This is a particular challenge in rapidly ageing societies such as China, which has a high prevalence of multimorbidity among older adults [5]. The proportion of the population with multimorbidity in China, as measured by population-based panel data, was 62% for people aged 50 years and 69% for those aged over 75 years [9]. In terms of the determinants of multimorbidity, current studies highlight a range of individual factors, including demographic (e.g., age, sex and race) and socio-economic (e.g., education and income) characteristics [9–12]. However, research on possible contextual and environmental determinants of multimorbidity is less common, and in particular the role of air pollution (AP) remains poorly understood [13].

Recent research on elderly health shows that older people are more susceptible to environmental factors than younger adults [14], with higher risks of living with chronic diseases due to exposure to environmental pollution [15, 16]. Furthermore, there is abundant evidence on the association between AP and individual chronic diseases, for example, cardiorespiratory disease [17], chronic obstructive pulmonary disease (COPD) [18], diabetes [19], heart disease [20], hypertension [21], and kidney diseases [22]. Although chronic diseases cluster due to shared biological or environmental risk factors [13], there is limited understanding of how AP might operate to promote accumulation of multiple chronic diseases.

Similar to many LMICs, AP is an important public health risk in China: in 2017, 81% of the Chinese population lived in regions which exceed the World Health Organisation Interim Target 1 (35 *μg*/m^3^) [23]. In particular, ambient AP was estimated to be responsible for over 850,000 deaths in China in 2017 [23]. Although ambient AP has decreased markedly in the last two decades, the older population of China has spent a large proportion of the life course experiencing historically high levels of AP exposure [24, 25]. China therefore suffers from a double burden of multimorbid ageing and AP, and understanding the association between them may be beneficial for development of strategies to prevent or manage chronic diseases in later life.

Evidence shows that multimorbidity prevalence is likely higher in some social groups because chronic diseases often cluster due to common risk factors, such as socioeconomic deprivation and environmental risks [13]. These risk factors for diseases clustering make it difficult to isolate the effects of AP from other factors of socioeconomic deprivation [26]. Therefore, motivations for this study are not only to understand the relationship between historic AP exposure and changes in multimorbidity, but also to explore individual-level characteristics that are associated with multimorbidity inequalities.

In this study, we analyse the associations between cumulative, historic exposure to AP as predictive of cross-sectional multimorbidity disease clusters and multimorbidity accumulation. We use large, prospective and nationally representative survey data, the China Health and Retirement Longitudinal Study (CHARLS), linked with historical satellite data on PM_2.5_ exposure over 15 years. Using this novel dataset, we address a research gap for longitudinal studies of multimorbidity and provide assessment of the associations between AP and the development of multimorbidity.

## Methods

### Study population

Data used in this study are from three waves of the China Health and Retirement Longitudinal Study (CHARLS 2011, 2013, 2015), which is a nationally representative longitudinal survey of the middle-aged and elderly population of China, consisting of persons aged 45 years or older, as well as their spouses when possible. CHARLS used computer-assisted in-person interviews to obtain samples through four-stage stratified sampling, with an overall response rate of 80.5% at the baseline. From June 2011 and March 2012, CHARLS conducted the baseline survey (wave 1) that included assessments of the social, economic, and health circumstances of 17,705 respondents from 28 provinces, 150 cities/counties/districts, 450 communities, and 10,257 households [27]. Following wave 1, two follow-up surveys were conducted in 2013 and 2015.

**Fig 1:**
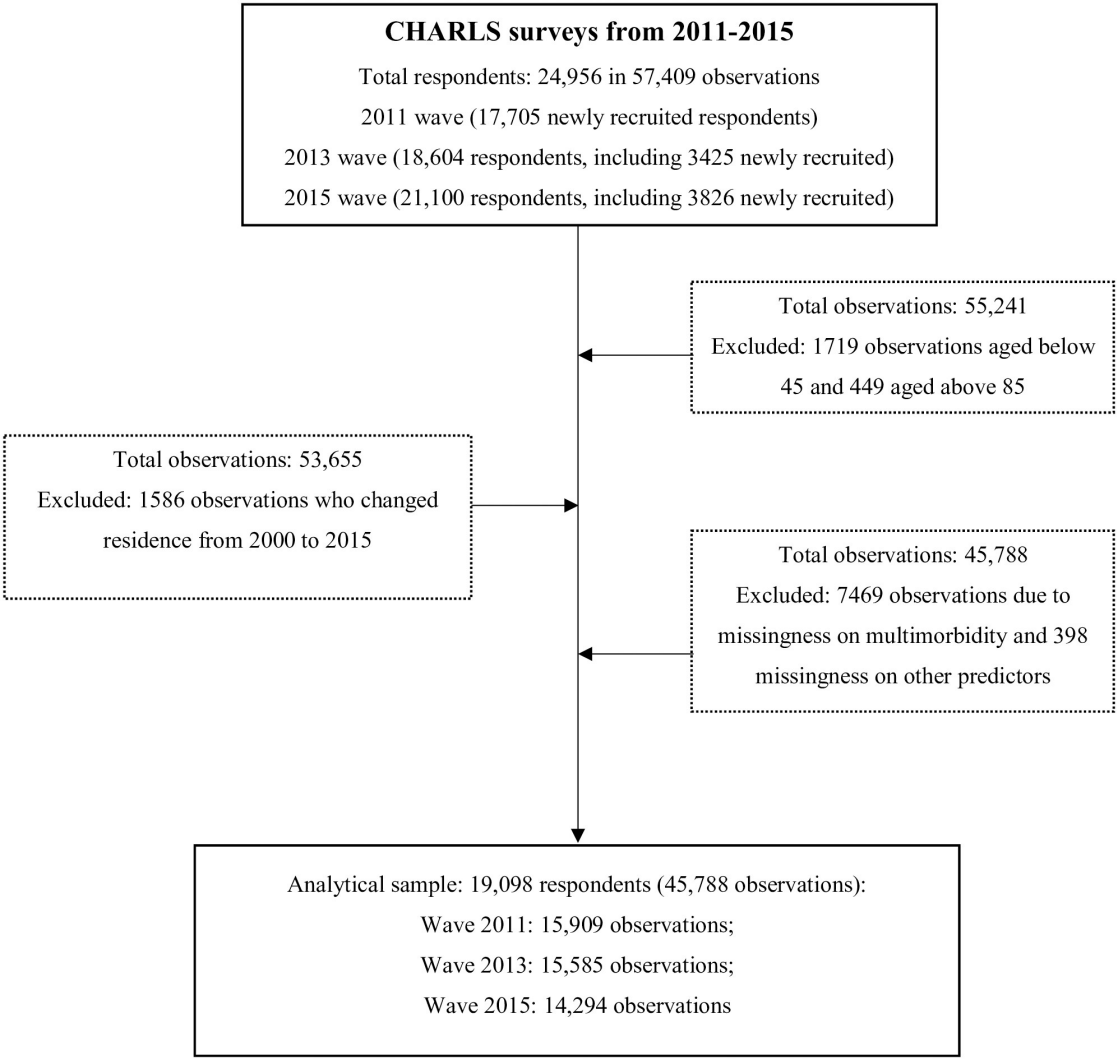
Flowchart of study inclusion criteria. Note: CHARLS, the China Health and Retirement Longitudinal Study.

Fig 1 shows the criteria of sample inclusion. In 2011, the baseline CHARLS sample size was 17,705. Between 2011 and 2013, 2,526 respondents attrited due to death (n = 441) or non-specified reasons (n = 2,125). In 2013, a refreshment sample was added of 3,425 new respondents, making the total 2013 sample consist of 18,604 individuals. The 2015 wave of CHARLS had a total sample size of 21,100, including 3,826 new joiners. Between 2013 and 2015, 689 respondents died and 1,658 attrited, and 1,017 respondents, interviewed in 2011 but missing in 2013, returned. It is noted that these refreshment samples did not participate in the first wave in 2011. Finally, the three waves of CHARLS include 24,956 respondents (57,409 observations).

We restrict our analysis to the 19,098 respondents (45,788 observations) from 125 cities who were aged 45 to 85 years old at any wave of the study. The listwise deletion process is also shown in Fig 1.

### Main outcome: Multimorbidity

The ‘Health status and function’ module in the CHARLS questionnaire includes 14 self-reported doctor-diagnosed chronic diseases for each respondent, asking “Have you been diagnosed with the following conditions by a doctor”: hypertension; dyslipidaemia; diabetes or high blood sugar; cancer or malignant tumour; chronic lung disease; liver disease; heart problems; stroke; kidney disease; stomach or other digestive diseases; emotional, nervous or psychiatric problems; memory-related disease; arthritis or rheumatism [12]. In line with previous CHARLS studies, we use a disease count approach where we summed 14 binary disease indicators (range 0–14) to capture multimorbidity [12, 28].

We exclude respondents who are missing any components of these 14 indicators of chronic disease. Using this method, there are 7,469 observations with missingness on multimorbidity. Note that CHARLS 2015 contributes to over half of these (4,654 observations), partly due to survey design, because around 2,800 respondents were interviewed in the Life History Survey (in 2014) but not in previous waves (CHARLS 2011 and 2013). In these cases, the respondents were not asked if they ever had a condition and therefore their chronic disease records are missing in 2015. We explain how we deal with missingness in the statistical analysis section.

### Air pollution: PM_2.5_

Many studies use ground air pollutants concentration (e.g., PM_2.5_, PM_10_, NO_2_) from monitoring stations to measure the exposure to AP [29, 30]. In China, however, most AP monitoring stations were established by the Ministry of Ecology and Environment only after 2013, limiting the ability to study long-term exposure.

Compared with ground monitoring data, satellite data with broad spatial coverage, a long-term data record, and high spatial resolutions could support the assessment of historical AP levels in developing regions. A detailed description of the ensemble machine learning model to generate our long-term PM_2.5_ exposure estimates is published elsewhere [31], and summarised briefly here. Given the large modelling domain, China was first divided into seven subregions using a geographically weighted regression approach to allow the machine learning algorithms to capture different spatiotemporal patterns of PM_2.5_ in each subregion caused by different terrain, weather conditions, and emission source profiles. A random forest, an extreme gradient boosting (XGBoost), and a generalized additive model (GAM) were then trained in each subregion. Their predictions of daily PM_2.5_ concentration levels at 10 km^2^ spatial resolution were combined using weights determined by prediction accuracy. Compared with previous models, this ensemble model provided more accurate out-of-range predictions at the daily and monthly levels. Based on the administrative regions in China, 150 cities of CHARLS are clustered to aggregate PM_2.5_ concentration from satellite data at the city level. To match with CHARLS, we selected monthly PM_2.5_ concentration as the temporal scale (see Table G in S1 File).

As used in previous studies, the measure of cumulative exposure is operationalised using the average mean of pollutant concentrations during the exposure window [32, 33]. Chinese ambient air quality standards use two cut-offs to indicate the hazardous level of exposures, 35 *μg*/m^3^ (Level 1) and 75 *μg*/m^3^ (Level 2) [34]. In this study, we exploit our longer-term PM_2.5_ data and calculate a more fine-grained measure: the average concentration of monthly exposure from March 2000 until the survey date, which provides a measure of historical exposure. Additionally, we initially categorise PM_2.5_ exposure into six groups using 10 *μg*/m^3^ intervals: 0–35, 36–45, 46–55, 56–65, 66–75, 76+ *μg*/m^3^. Due to small numbers exposed to PM_2.5_ over 76+ *μg*/m^3^, we later collapse the last two categories resulting in five groups.

### Covariates: Demographic, socioeconomic status (SES), health behaviour and regional factors

In this study, the covariates include four components: demographic, socioeconomic status (SES), behavioural, and contextual factors. Demographic variables include age, age squared, gender, and marital status (single vs. partnered). Individual SES consists of education (no schooling, primary, middle or more education), occupation (agricultural, non-agricultural, and managerial), and HuKou (rural, rural-urban, urban). CHARLS life history survey records the longest occupation during the respondents’ occupational history. Agricultural jobs include farming, fishing, managing forest products or fruit trees, raising livestock, and selling these products in the market. Non-agricultural jobs include civil servants, office clerks or non-agricultural self-employment (e.g., running a restaurant or supermarket). Respondents who are in a supervisory position in their offices are considered “managers”. Respondents who have never worked (e.g., “housewife”, or disabled people) are sparse in the CHARLS (only 137 respondents) and are marked as missing.

HuKou is a special national household registration system in China that has two categories: rural and urban. People usually remain in the same HuKou as their parents, and once HuKou is registered, it is difficult to change even if people move. HuKou is related to occupational status, education, and health care access [35, 36]. Due to the urbanisation and internal migration, people who originate in a rural HuKou increasingly live in the urban areas. Thus, considering both HuKou and current residence, there are three types: rural (rural HuKou living in rural areas), rural-urban (rural HuKou living in urban areas), and urban (urban HuKou living in urban areas). HuKou is an important feature in this study as it is strongly related to personal SES, not solely housing address [35].

Smoking status is controlled for as an important health behaviour (never smoking, former smoker, and current smoker). To account for the urbanisation and industrialisation of cities, this study includes annual regional Gross Domestic Product (GDP) at the city level (logged).

### Analysis strategies

To analyse multimorbidity disease clusters, we use latent class analysis (LCA) on a cross-sectional sample of the baseline wave of CHARLS as LCA can pick up clusters of diseases shared with the common risk factors. The LCA models can identify multimorbidity patterns by assigning individuals to a set of discrete, mutually exclusive groups—latent classes—based on their responses to the 14 chronic disease indicators in the CHARLS. A sequence of 14 LCA models was estimated starting with a one-class model and increasing the number of classes in a stepwise approach. Following examples in previous studies [37, 38], the LCA model selection was based on examinations of several fit indices, including AIC, BIC and likelihood estimates. We then regressed the resulting latent class memberships on cumulative PM_2.5_ exposure in 2011 using multinomial regression, adjusting for the covariates discussed above.

We use growth curve modelling (GCM) to examine the relationship between PM_2.5_ exposure in the period of 2000–2015 and multimorbidity accumulation between 2011–2015. An important advantage of GCM is the ability to model the trajectories of individuals over time and distinguish within-individual from between-individual heterogeneities in estimating multimorbidity accumulation/changes shaped by other variables [39]. In this study, we use three waves of CHARLS across four years (2011–2015) of data collection. As multimorbidity is a count variable, we assume a Poisson distribution. As there is likely a non-linear relationship between PM_2.5_ exposure and elderly health, we add a quadratic term of PM_2.5_ exposure, as well as alternative models (detailed below) using categorisations of PM_2.5_. To examine heterogeneity in the associations of PM_2.5_ exposure among different groups, we further explore the interactions between PM_2.5_ exposure and age, SES (education, occupational status, HuKou-residence), and smoking status.

We conduct a number of robustness checks. First, we run the same set of LCA and GCMs models but use a categorical measure of PM_2.5_ exposure to allow for a more flexible estimation of the association between AP and multimorbidity (shown in Tables B and C in S1 File). Second, we run the GCMs first on the entire sample 45–85 years, then we subset the data into ages 45–64 and 65–85 years to compare middle-aged and oldest individuals (Tables D and E in S1 File). Third, given that 7,867 observations are deleted due to missingness, we apply multiple imputation (MI) using chained equations to complete our analysis samples under the missing-at-random assumption. We then use multilevel random-intercept Poisson regression to compare the results from the MI and complete datasets (Table F in S1 File).

## Results

### Descriptive analysis

At baseline, the average age of respondents is approximately 59 years old. 49% are men. 39% of the population attained primary education and 34% had middle or higher education. 78% of respondents are registered with rural HuKou but 19% respondents with rural HuKou are living in urban areas; 72% worked for agricultural jobs, and 88% are married. Average multimorbidity is 1.5 at baseline, increasing to 2.08 by 2015. From 2011 to 2015, average PM_2.5_ rises only slightly from 51.27 to 52.90 *μg*/m^3^ (Table 1). In addition, Table 1 shows that descriptive statistics for both the baseline and entire period’s analytical samples are very similar to the entire sample, suggesting that sample selection, including attrition, may not substantially affect results.

**Table 1:** Descriptive statistics of the study population in CHARLS 2011, 2013, 2015.

	CHARLS	CHARLS	CHARLS	CHARLS 2011-	CHARLS
	2011 (analysis)	2013 (analysis)	2015 (analysis)	2015 (analysis)	2011–2015
Multimorbidity (Mean/SD)	1.45 (1.43)	1.60 (1.52)	2.08 (1.75)	1.69 (1.59)	1.69 (1.59)
Cumulative PM_2.5_ exposure (Mean/SD)	51.27 (15.63)	52.10 (16.44)	52.90 (16.84)	52.06 (16.31)	52.01 (16.42)
Age (Mean/SD)	59.10 (9.49)	60.01 (9.54)	61.32 (9.26)	60.10 (9.48)	59.82 (9.66)
Gender (N/%)					
*Men*	7,795 (49.00)	7,582 (48.65)	6,887 (48.18)	9,277 (48.58)	11,358 (49.12)
*Women*	8,114 (51.00)	8,003 (51.35)	7,407 (51.82)	9,821 (51.42)	11,766 (50.88)
Education (N/%)					
*No schooling*	4,318 (27.14)	4,019 (25.79)	3,621 (25.33)	4,914 (25.73)	5,572 (24.14)
*Primary*	6,243 (39.24)	6,259 (40.16)	5,824 (40.74)	7,548 (39.52)	9,026 (39.11)
*Middle +*	5,348 (33.62)	5,307 (34.05)	4,849 (33.92)	6,636 (34.75)	8,481 (36.75)
HuKou (N/%)					
*Rural*	9,320 (58.58)	9,193 (58.99)	8,481 (59.33)	10,852 (56.21)	12,647 (54.18)
*Rural-urban*	3,065 (19.27)	2,973 (19.08)	2,734 (19.13)	3,777 (19.56)	4,757 (20.38)
*Urban*	3,524 (22.15)	3,419 (21.94)	3,079 (21.54)	4,676 (24.22)	5,938 (25.44)
Occupation (N/%)					
*Agricultural*	11,413 (71.74)	11,965 (76.77)	10,661 (74.58)	14,276 (67.80)	16,649 (66.55)
*Non-agricultural*	3,582 (22.52)	3,4015 (21.82)	2,917 (20.41)	5,146 (24.44)	6,371 (25.47)
*Managerial*	914 (5.75)	219 (1.41)	716 (5.01)	1,633 (7.76)	1,998 (7.99)
Marital (N/%)					
*Partnered*	13,976 (87.85)	13,660 (87.65)	12,420 (86.89)	16,960 (85.52)	20,589 (85.96)
*Single*	1,933 (12.15)	1,925 (12.35)	1,874 (13.11)	2,871 (14.48)	3,362 (14.04)
Smoking status (N/%)					
*Never*	9,433 (59.29)	8,869 (56.91)	8,317 (58.19)	11,774 (53.62)	14,119 (53.92)
*Former*	1,401 (8.81)	1,146 (7.35)	1,964 (13.74)	3,176 (14.46)	3,721 (14.21)
*Current*	5,075 (31.90)	5,570 (35.74)	4,017 (28.07)	7,007 (31.91)	8,345 (31.87)
Log GDP (Mean/SD)	10.29 (0.56)	10.47 (0.65)	10.47 (0.63)	10.41 (0.63)	10.42 (0.62)
Number of respondents	15,909	15,585	14,294	19,098	23,124

### Latent class analysis

First, we use LCA to explore the association between PM_2.5_ exposure and multimorbidity patterns at baseline (CHARLS 2011). Based on the comparisons of AIC, BIC, and likelihood estimates, the four-class model was chosen as the final model in this study (Table A and Fig A in S1 File). Table 2 shows the distribution of the sample across the four classes. Based on the probability distribution of chronic diseases across the classes, they are labelled: respiratory (Class 1), musculoskeletal (Class 2), cardio-metabolic (Class 3) and relatively healthy (Class 4). The label takes its name from the main diseases (items) that characterise it. For example, we labelled Class 1 as respiratory because the probability of lung diseases is 0.79, which means 79% of respondents in Class 1 suffered from lung diseases. Similarly, labels of class 2 and 3 are originated from high probabilities of arthritis/rheumatism (0.81) and hypertension (0.71).

**Table 2:** Class proportions and class-specific probabilities from a four-latent-class model of chronic conditions.

	Latent Class			
	Class 1	Class 2	Class 3	Class 4
**Assigned label**	Respiratory	Musculoskeletal	Cardio-metabolic	Relatively healthy
Class Proportion	0.075	0.207	0.172	0.546
**Items (chronic diseases)**	**Response probabilities**			
Hypertension	0.319	0.243	**0.713**	0.129
Dyslipidaemia	0.099	0.039	0.394	0.029
Diabetes	0.069	0.027	0.244	0.021
Cancer/malignant tumour	0.011	0.006	0.034	0.005
Lung diseases	**0.787**	0.094	0.053	0.034
Liver diseases	0.072	0.065	0.052	0.018
Heart problems	0.269	0.156	0.354	0.025
Stroke	0.044	0.024	0.102	0.005
Kidney diseases	0.123	0.122	0.088	0.023
Stomach/digestive diseases	0.323	0.447	0.204	0.138
Emotional/nervous/psychiatric	0.041	0.018	0.017	0.011
Memory-related diseases	0.059	0.020	0.051	0.005
Arthritis/rheumatism	0.477	**0.813**	0.340	0.155
Asthma	0.496	0.018	0.017	0.006

### Multinomial regression

Second, we present the results from the cross-sectional multinomial models analysing the associations between PM_2.5_ and multimorbidity patterns at baseline, controlling for a set of covariates. The findings show that higher exposure to AP is associated with a higher prevalence of the other three classes of chronic diseases, compared to those who are “relatively healthy” (Table 3). Specifically, a 1 *μg*/m^3^ increase in cumulative exposure to PM_2.5_, is associated with a higher likelihood of belonging to respiratory, musculoskeletal and cardio-metabolic cluster: 2.4% (95% CI: 1.02, 1.03), 1.5% (95% CI: 1.01, 1.02) and 3.3% (95% CI: 1.03, 1.04), respectively.

**Table 3:** Odds ratios with 95% confidence intervals from multinomial logistic regression results for PM_2.5_ by latent disease class (reference class: Relatively healthy), CHARLS 2011.

	Class 1:	Class 2:	Class 3:
	Respiratory	Musculoskeletal	Cardio-metabolic
PM_2.5_ exposure	1.024***	1.015***	1.033***
	(1.017–1.031)	(1.011–1.020)	(1.026–1.039)
Age	1.046***	0.971***	1.041***
	(1.033–1.058)	(0.963–0.980)	(1.030–1.052)
Gender (ref: Men)			
*Women*	0.479***	0.427***	0.682**
	(0.352–0.652)	(0.347–0.524)	(0.522–0.890)
Education (ref: no schooling)			
*Primary*	0.963	0.671***	0.989
	(0.751–1.235)	(0.569–0.791)	(0.788–1.241)
*Middle +*	0.879	0.995	1.481**
	(0.629–1.227)	(0.801–1.235)	(1.115–1.967)
HuKou (ref: Rural)			
*Rural-urban*	0.829	1.003	1.294**
	(0.631–1.088)	(0.846–1.189)	(1.032–1.622)
*Urban*	2.132***	1.766***	4.695***
	(1.520–2.991)	(1.352–2.307)	(3.507–6.285)
Occupation (ref: agricultural)			
*Non- agricultural*	1.456**	1.287*	1.575***
	(1.097–1.934)	(1.038–1.596)	(1.230–2.018)
*Managerial*	0.929	1.669*	1.277
	(0.490–1.763)	(1.125–2.477)	(0.782–2.086)
Marital (ref: partnered)			
*Single*	1.408*	1.176	1.000
	(1.064–1.863)	(0.951–1.455)	(0.766–1.305)
Smoking (ref: never)			
*Former*	2.820***	0.762#	1.477*
	(1.945–4.088)	(0.556–1.045)	(1.033–2.111)
*Current*	1.467**	0.966	0.691**
	(1.100–1.956)	(0.792–1.178)	(0.536–0.892)
Log GDP	1.488***	2.393***	2.686***
	(1.220–1.816)	(2.096–2.733)	(2.265–3.186)
Constant	0.0001***	0.002***	3.93e-07***
	(0.000–0.001)	(0.0004–0.006)	(0.000–0.000)

*** p<0.001,

** p<0.01,

* p<0.05,

^#^ p<0.1

In terms of the other covariates, the regression model shows that women have a lower likelihood of belonging to any of three multimorbidity classes compared with the healthy class. Older age is associated with a higher likelihood of belonging to respiratory and cardio-metabolic clusters but with a lower likelihood in musculoskeletal cluster. The relationship between education and multimorbidity patterns is complex. Compared with respondents without schooling, those with primary education have a lower likelihood of belonging to the musculoskeletal cluster, and those with middle or higher education have a higher likelihood of belonging to cardio-metabolic cluster.

Respondents with urban HuKou have a higher likelihood of belonging to any of the three disease clusters, especially the cardio-metabolic cluster. Working in non-agricultural positions is associated with a higher likelihood of being in those three classes. Being single is associated with a higher likelihood of being in the respiratory cluster but is not associated with membership in the musculoskeletal and cardio-metabolic clusters. Smoking status is associated with a higher likelihood of respiratory and cardio-metabolic clusters but not associated with the musculoskeletal cluster. Compared with non-smokers, former smokers have a higher likelihood of belonging to the cardio-metabolic cluster, whereas current smokers have a lower likelihood. Higher GDP is associated with a higher likelihood of belonging to any of the three disease clusters.

### Heterogeneity in patterns

**Fig 2:**
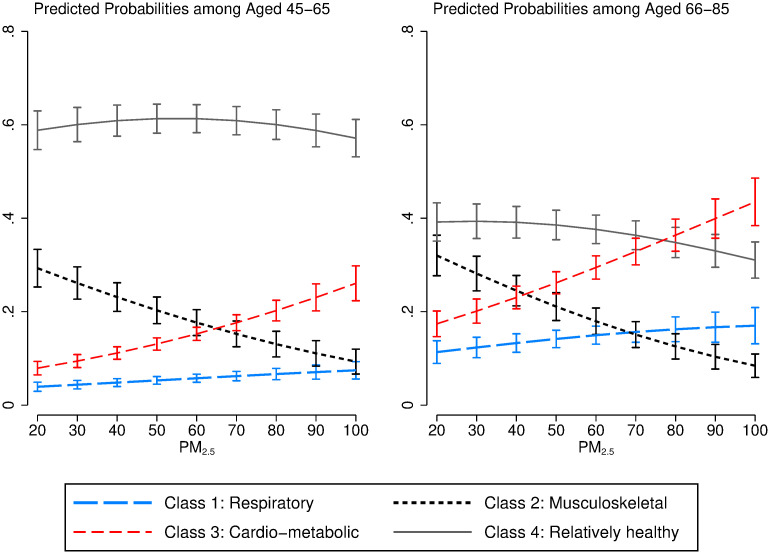
Predicted probabilities of 11-year PM_2.5_ exposure on latent multimorbidity patterns by age groups. Note: Models adjusted for age, age squared, gender, education, HuKou-residence, occupations, marital status, smoking status and logged GDP.

We also analyse the heterogeneity in multimorbidity cluster membership by age, gender and HuKou. Fig 2 shows the association between multimorbidity patterns and 11-year exposure to PM_2.5_, comparing middle-aged (45–65 years old) with older adults (66–85 years old). Generally, higher exposure to PM_2.5_ is associated with a higher probability of belonging to respiratory and, especially, cardiometabolic clusters. Unexpectedly, lower levels of PM_2.5_ are associated with higher likelihood of belonging to the musculoskeletal cluster. We can see the negative associations of PM_2.5_ exposure are more substantial among the group aged 66–85, because there is a lower likelihood of belonging to the relatively healthy class but a higher likelihood in the respiratory, musculoskeletal, and cardio-metabolic clusters when PM_2.5_ exposure levels are elevating.

### Growth curve models

To examine the associations between cumulative PM_2.5_ exposure and multimorbidity accumulation, we conduct a set of GCMs. First, we examine the linear and non-linear relationships between PM_2.5_ exposure and multimorbidity by adding linear and quadratic terms of PM_2.5_ exposure in GCMs (Table 4). The significant coefficients of both PM_2.5_ exposure and its quadratic term suggest a u-shaped association between PM_2.5_ exposure and multimorbidity (Table 4). This means that exposure to PM_2.5_ is positively associated with the likelihood of multimorbidity when the concentration of PM_2.5_ exposure is higher than 53.3 *μg*/m^3^. The inflection point of the u-shaped curve is slightly lower among older adults (Tables D and E in S1 File). For example, the inflection point is 56 *μg*/m^3^ among adults aged 45–64 years old and declines to 43 *μg*/m^3^ among adults aged 65–85 years old, suggesting that higher AP exposure has worse health effects on the older population.

**Table 4:** Coefficients with 95% confidence intervals of growth curve models on the associations of 10 *μg*/m^3^ increase in PM_2.5_ exposure and its quadratic terms and covariates, with multimorbidity, CHARLS 2011–2015.

	Model 1:	Model 2: Model 1	Model 3: Model 2	Model 4: Model 3
	Base	+ Education	+ SES	+ Smoking+ GDP
PM_2.5_ exposure	-0.191***	-0.200***	-0.206***	-0.202***
	(-0.235 - -0.147)	(-0.244 - -0.155)	(-0.250 - -0.162)	(-0.246 - -0.158)
PM_2.5_ exposure square	0.018***	0.019***	0.019***	0.019***
	(0.014–0.022)	(0.015–0.023)	(0.015–0.023)	(0.015–0.023)
Age	0.140***	0.139***	0.137***	0.137***
	(0.124–0.155)	(0.123–0.154)	(0.121–0.153)	(0.122–0.153)
Age square	-0.0009***	-0.0009***	-0.0009***	-0.0009***
	(-0.0011 - -0.0008)	(-0.00099 - -0.0007)	(-0.00098 - -0.0007)	(-0.00099 - -0.0007)
Gender (ref: Men)				
*Women*	0.152***	0.189***	0.180***	0.197***
	(0.124–0.180)	(0.159–0.219)	(0.150–0.210)	(0.159–0.235)
Education (ref: no schooling)				
*Primary*		0.147***	0.136***	0.135***
		(0.110–0.184)	(0.0983–0.173)	(0.098–0.173)
*Middle +*		0.130***	0.0867***	0.0869***
		(0.089–0.171)	(0.0423–0.131)	(0.0425–0.131)
HuKou (ref: Rural)				
*Rural-urban*			-0.029	-0.025
			(-0.066–0.008)	(-0.062–0.012)
*Urban*			0.105***	0.107***
			(0.066–0.144)	(0.068–0.146)
Occupation (ref: agricultural)				
*Non- agricultural*			-0.008	-0.012
			(-0.043–0.026)	(-0.046–0.022)
*Managerial*			-0.042	-0.044
			(-0.095–0.011)	(-0.097–0.009)
Marital (ref: partnered)				
*Single*			0.014	0.017
			(-0.024–0.052)	(-0.021–0.054)
Smoking (ref: Never)				
*Former*				0.175***
				(0.137–0.214)
*Current*				-0.024
				(-0.060–0.012)
Log GDP				-0.014
				(-0.036–0.008)
Constant	-4.518***	-4.631***	-4.521***	-4.385***
	(-5.015 - -4.022)	(-5.131 - -4.131)	(-5.024 - -4.018)	(-4.931 - -3.838)
**Random effects**				
Variance				
*Individuals (age)*	4.75e-17***	3.12e-17***	2.23e-17***	4.84e-17***
*Years*	0.608***	0.607***	0.602***	0.593***
	(0.586–0.631)	(0.585–0.630)	(0.580–0.625)	(0.572–0.615)
Covariance				
*Individuals—Years*	3.95e-12***	2.85e-12***	2.49e-12***	4.87e-12***
Log likelihood	-69877.261	-69845.32	-69824.071	-69750.266
Observations	45,788	45,788	45,788	45,788
Number of IDs	19,098	19,098	19,098	19,098

*** p<0.001,

** p<0.01,

* p<0.05,

^#^ p<0.1

In Model 4 (the full model) women have a higher prevalence of multimorbidity than men, and there is a curvilinear increase in multimorbidity over age. Unexpectedly, people with higher education and urban HuKou have a higher prevalence of multimorbidity. Occupation and partnership status is not significantly associated with the risk of multimorbidity. Compared with respondents who never smoked, former smokers have a higher prevalence of multimorbidity, but current smokers do not. There is not a significant association between GDP and multimorbidity accumulation.

### Heterogeneity in multimorbidity accumulation

**Fig 3:**
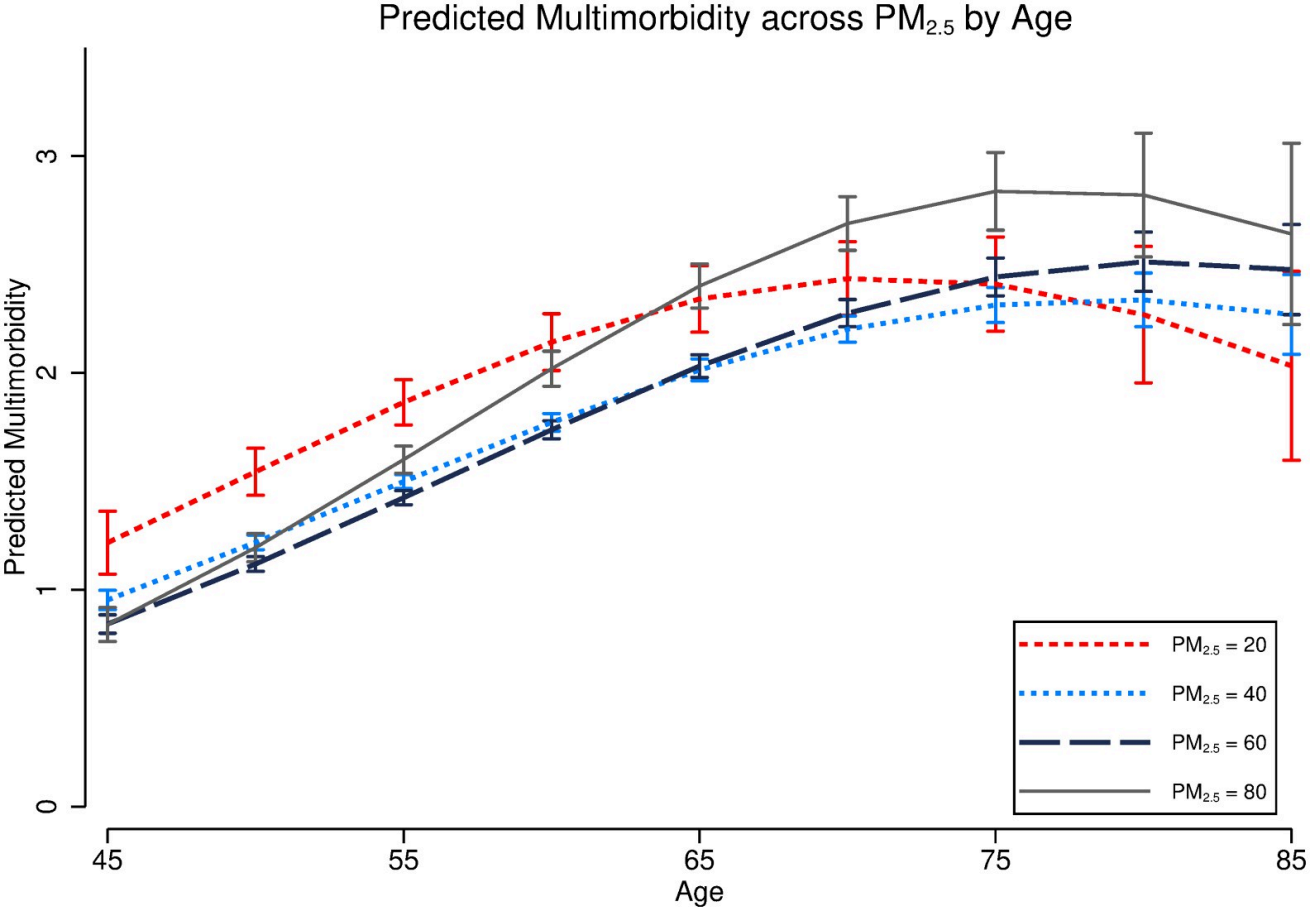
Predicted multimorbidity score across PM_2.5_ exposure by age. Note that this model controls age, age squared, gender, education, HuKou-residence, occupations, marital status, smoking status, and logged GDP.

To explore the trajectory of multimorbidity associated with PM_2.5_ exposure across age, based on Model 4 in Table 4, we interact age with PM_2.5_ exposure and PM_2.5_ squared; then, we plot the association between PM_2.5_ exposure and multimorbidity scores in Fig 3. Generally, respondents exposed to higher PM_2.5_ exposure have higher risk of multimorbidity. Over the age of 60, the respondents in the highest AP exposure categories (e.g., PM_2.5_ over 80+) have steeper multimorbidity trajectories than those in lower exposure categories. However, it is only at certain older ages (75 years, for example) that this is statistically significant. There is an inverted u-shaped relationship between age and multimorbidity, indicating a higher risk in multimorbidity with ageing among adults aged under 75 but a decline in multimorbidity with ageing among those between 75–85 years old. The oldest old (aged over 75) have a lower prevalence of multimorbidity.

**Fig 4:**
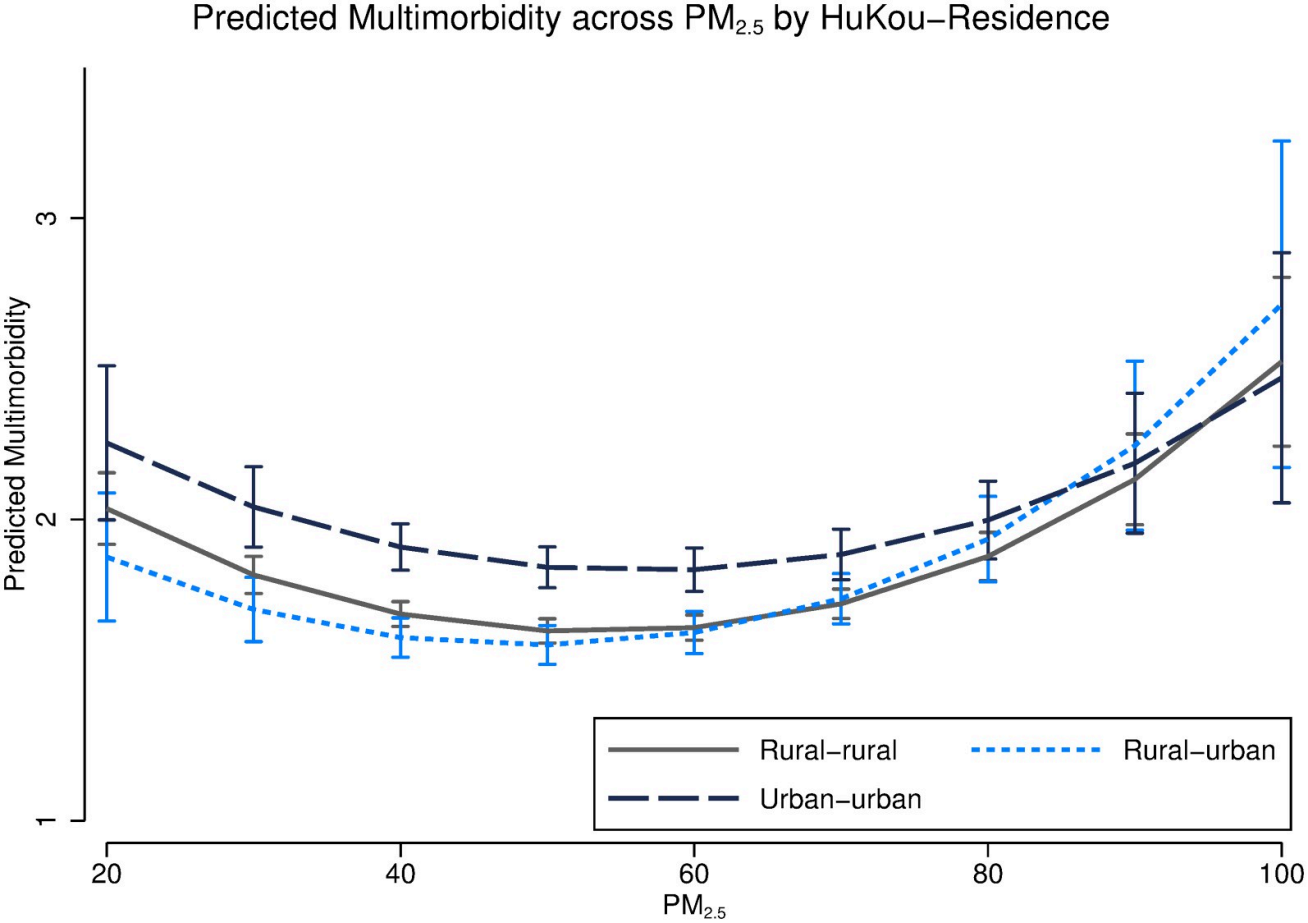
Predicted multimorbidity score across PM_2.5_ exposure by HuKou-residence. Note that this model controls age, age squared, gender, education, HuKou-residence, occupations, marital status, smoking status and logged GDP.

We conduct a set of analyses to understand heterogeneities in the association between PM_2.5_ and multimorbidity among different HuKou-residence groups. Generally, we can see that there is a u-shaped relationship between PM_2.5_ exposure and multimorbidity accumulation among all groups. Fig 4 shows that people with urban HuKou have a higher likelihood of being multimorbid than those with a rural Kukou when PM_2.5_ concentration is lower than 70 *μg*/m^3^, but when PM_2.5_ exposure is over 70 *μg*/m^3^, the associations of PM_2.5_ are not different between rural, rural-urban and urban residents. However, respondents with rural HuKou share a similar trajectory of multimorbidity across PM_2.5_ exposure regardless of their residences.

We conduct a number of robustness checks. We use categorical PM_2.5_ exposure to check the non-linear relationship between PM_2.5_ exposure and multimorbidity. First, we find that compared with the relatively healthy class, higher exposure to PM_2.5_ is associated with a higher prevalence of the other three classes of chronic diseases (Table B in S1 File). Second, higher exposure to PM_2.5_ is associated with a higher incidence rate ratio of multimorbidity in the longitudinal analyses (Table C in S1 File). In addition, comparing findings from complete data and MI data shows that results are consistent (Table F in S1 File). These sensitivity analyses indicate the robustness of our results.

## Discussion

By linking the CHARLS, a nationally representative dataset, with historical PM_2.5_ records derived from remote sensing technology, we investigate the associations between long-term exposure to PM_2.5_ and patterns and accumulation of multimorbidity. Incorporating 15-year PM_2.5_ exposure histories enables us to capture the associations between long-term exposure and chronic disease accumulation. To the best of our knowledge, this is the first study to establish a link between PM_2.5_ exposure and multimorbidity patterns, and to estimate associations between cumulative exposure and the accumulation of multimorbidity longitudinally. Findings from the LCA for multimorbidity patterns suggest that higher exposure to PM_2.5_ is associated with a higher risk of cardio-metabolic and respiratory multimorbidity (dominated by lung disease), whereas lower PM_2.5_ exposure is associated with a higher likelihood of musculoskeletal multimorbidity. Our longitudinal GCM findings show that both lower and higher historical AP exposure is associated with faster multimorbidity accumulation. This u-shaped association may be explained by the different multimorbidity clusters at opposite ends of AP exposure spectrum, as shown in the LCA models. These estimates suggested that for many middle-income countries such as China, more efforts to reduce PM_2.5_ concentrations would be associated with a substantial reduction in burden of multiple diseases.

First, our LCA analyses show that the four latent classes are differentially associated with PM_2.5_ exposure, which are partly in accordance with previous studies of AP and single diseases [40, 41]. For example, higher exposure to PM_2.5_ is associated with an increased likelihood of developing respiratory diseases and particularly, cardio-metabolic diseases (a cluster dominated by hypertension). Previous studies about associations between AP and hypertension are inconsistent, some of which find significant associations between them but others do not [32, 42–44]. These studies suggest that hypertension may be related to AP or caused and exacerbated by other cardiometabolic disorders (e.g., cardiovascular diseases) attributable to PM_2.5_.

One unexpected finding, partly inconsistent with previous research [45], is that higher PM_2.5_ exposure is associated with a reduced likelihood of developing musculoskeletal diseases such as arthritis, or to put it another way, that those suffering with musculoskeletal multimorbidity are more likely to live in areas of low air pollution. A possible explanation is that the rural-urban difference in environmental exposures (besides PM_2.5_ exposure)—like noise, green space, food environments—which makes urban residents more likely to be ill from metabolic conditions than achy joints and cartilages [32, 46–48]. Second, rural and urban residents may have had different occupational exposures throughout their lifetime. Our analysis shows that there are more urban residents working at non-agricultural jobs than rural residents (65% vs. 15%). It is likely that urban residents have led a more sedentary lifestyle (working in white-collar occupations) compared with rural residents who have been employed in farming, forest, hunting and fishing that are physically demanding [49]. This might predispose them to develop musculoskeletal conditions as there is more wear and tear on the body [50, 51]. Third, although AP exposure is higher in urban areas than in rural areas in China, there are potential offsetting benefits of urban residence, such as better access to health care [52]. Urban residents may be more likely to have better healthcare and get diagnosed for conditions that are not immediately obvious (e.g., hypertension); hence, those disorders might be undiagnosed and potentially underestimated in rural residents. Fourth, in China, rural areas consume more solid fuels (e.g., coal and wood) as the major source of energy [53], which leads to more severe indoor air pollution that is associated with increased odds of arthritis [54]. Furthermore, compared with respiratory or cardio-metabolic diseases, musculoskeletal diseases (especially arthritis) might be more influenced by health care than AP [55]. This should be explored in future research by using finer measures of rural-urban residence and migration, and by explicitly investigating these potential mechanisms for disparities.

Our results of GCMs suggest that cumulative PM_2.5_ exposure is associated with higher multimorbidity scores at both lower and higher levels of PM_2.5_ (e.g., a u-shaped association). Although there are few studies related to the u-shape association of environmental pollution with multimorbidity, the u-shape links between AP and health risks (e.g., hospital admissions and mortality) are well established [56]. Most AP in Chinese cities comes from industrial production and vehicle traffic, which is increasing in conjunction with economic development [57]. Due to a high proportion of industrial sectors and increasing traffic intensity in China, massive fossil fuels, especially coal, are consumed for economic development, and AP has been more and more severe [57]. This may explain why higher AP is associated with higher risk of chronic health diseases. Apart from the above contextual characteristics (urbanisation and economic development), the elevated multimorbidity at lower PM_2.5_ exposure might be attributed to higher levels of musculoskeletal multimorbidity that are related to the differences in the rural-urban context (as explained above). However, due to the strong side effects of AP, higher exposure to PM_2.5_ could lead to increased risk of multimorbidity once PM_2.5_ levels exceed the threshold (approximately 53 *μg*/m^3^ in our findings). The annual average PM_2.5_ exposure in China in 2015 was 55.2 *μg*/m^3^ [58], indicating that current PM_2.5_ exposure is harmful to human health among the majority of Chinese adults. These findings suggest that corresponding policies regarding AP should be implemented based on the strategies of sustainable development and disease prevention.

Third, the associations of multimorbidity accumulation show unexpected links with SES (the higher SES, including higher education and urban HuKou, is associated with a higher risk of multimorbidity). These unexpected results regarding SES can be understood from two perspectives. First of all, respondents with higher SES are more likely to be urban dwellers, who are exposed to higher AP as well as engaging in less physical exercise and more harmful health behaviours [59, 60]. Second, as mentioned above, this rural-urban differences in social fabric, including contextual and compositional factors associated with rural-urban residence, are perhaps fully captured by the current covariates. For example, urban residents tend to have higher educational levels and other social advantages (e.g., income, wealth, health awareness, social support etc.), as well as better access to health care. In addition, the measure of multimorbidity in this study is based on self-rated diagnosis, so respondents with higher SES might report a higher prevalence of chronic diseases [59]. The rural-urban environmental context may also contribute to these findings. Urban residents experience more environmental stressors (including noise and fast food), which lead to higher risks of multimorbidity [61, 62].

In the LCA results, we find that women have a lower likelihood of belonging to multimorbid classes, which is inconsistent with our findings in the longitudinal analyses. The reason might be that the multimorbidity classes in the LCA are each dominated by one disease. In this study, the respiratory cluster is dominated by lung diseases, and cardio-metabolic and musculoskeletal clusters are dominated by hypertension and arthritis. These findings are in line with previous studies that show that the prevalence of pulmonary diseases, hypertension and arthritis, is higher in men than women in China [63–65]. However, when considering multiple chronic diseases, women might have higher risks in multimorbidity because Chinese women have less access to medical resources than men [12].

In additional analyses (Figs 3 and 4), we further explore the associations between PM_2.5_ exposure and multimorbidity accumulation by age and HuKou-residence. First, these associations by age show that PM_2.5_ is associated with a higher number of morbidities among respondents aged 45–75, whereas for respondents aged over 75, PM_2.5_ is associated with lower risk of multimorbidity. This might be due to mortality selection. Older people with multiple morbidities might die younger or they are less exposed to AP. Second, we see that when PM_2.5_ exposure is lower than 80 *μg*/m^3^, respondents with urban HuKou have a higher risk of multimorbidity accumulation than those with rural residence. As previously discussed, urban residents have more advantageous socio-economic conditions and might report a higher prevalence of multimorbidity. However, when PM_2.5_ exposure exceeds 80 *μg*/m^3^, there is no significant difference in the associations between PM_2.5_ exposure and multimorbidity among groups with different HuKou-residence. This inconsistent finding might be due to two reasons. Firstly, the insignificant difference might be due to small sample sizes. There are only 5% of respondents living in cities where PM_2.5_ levels are over 80 *μg*/m^3^. Secondly, due to more opportunities in major cities (e.g., more high-paying jobs, better education, and more access to health care), many people with rural HuKou decide to live and work in urban areas (30% people with rural HuKou live in urban areas). This might explain why there is no rural-urban difference in areas with high exposure (over 80 *μg*/m^3^).

Our study has several advantages over previous studies. First, we link the CHARLS with historical PM_2.5_ records over a 15-year period, which enables us to measure long-term exposure to AP for each respondent. Second, this is a first study to analyse associations between AP and multimorbidity clusters. Nevertheless, there are several limitations in this study. First, we were unable to obtain detailed addresses of respondents from CHARLS, and thus we use city-level exposure to predict individual multimorbidity. In the Chinese context, a city might cover a large area (e.g., Beijing city) and consist of inner-city areas (more urban, more polluted) and suburb areas (more rural, less polluted). This means that we cannot accurately compare the variations within the same city. Future research should link AP at a smaller geographic scale. Second, multimorbidity is measured by self-reported doctor’s diagnosis which might underestimate chronic diseases due to lower levels of diagnosis in some groups [66]. This might be related to individual characteristics (e.g., gender, age) making it less likely to seek treatment or related to reduced access to healthcare in some places. Third, our findings only indicate the associations of PM_2.5_ with multimorbidity, and thus do not have a causal interpretation. We cannot rule out that our findings might be the result of other air pollutants (e.g., indoor air pollution, NO_2_, O_3_, etc), or contextual factors that are highly correlated with PM_2.5_ exposure, for example, lack of green space or noise pollution. Fourth, our latent class analysis is based on 14 chronic diseases available in the CHARLS and does not therefore cover all chronic diseases. From the perspective of the association between multimorbidity and exposure to air pollution, the breadth of chronic diseases not included in the CHARLS survey makes it difficult to predict the direction of bias for each cluster. Thus, further analyses should collect a broader range of diseases to reduce the bias in disease clusters. Fifth, 13% of participants (7,469 observations) did not have complete disease-reporting data. In longitudinal analyses, we used multiple imputation to complete the dataset; however, for the LCA analyses, this was not an option. It is possible that underreporting of certain types of diseases might create a bias in how disease clusters are associated with exposure to air pollution, although it is difficult to predict that bias. Future research with more complete disease data could help to solve this issue. Finally, we measure multimorbidity accumulation over a relatively short period of 4 years (2011–2015).

## Conclusion

This study provides evidence showing that higher cumulative exposure to PM_2.5_ is associated with increased risks of all types of multimorbidity patterns, but especially cardio-metabolic multimorbidity, and higher multimorbidity accumulation over 15 years. Notably, areas with low AP exposure still have higher rates of multimorbidity, associated with musculoskeletal disorders. Thus, our study highlights how multimorbidity clusters vary contextually and reveals that PM_2.5_ exposure is more detrimental to health among older adults. However, further research is needed to unpick the nexus of contextual and compositional factors associated with the development of chronic diseases in rural and urban settings and to detect their causal mechanisms.

## Supporting information

S1 File (DOCX)

S1 Data Monthly remotely-sensed PM_2.5_ records from 2000 to 2015. (CSV)
