# Supplementary material for: A longitudinal analysis of PM2.5 exposure and multimorbidity clusters and accumulation among adults aged 45-85 in China
Source: PLOS Glob Public Health. 2022 Jun 29;2(6):e0000520. doi: 10.1371/journal.pgph.0000520 (PMC10021527; doi:10.1371/journal.pgph.0000520)
Supplement: S1 File — (DOCX) [file pgph.0000520.s001.docx]

**Supplemental material**

Table A. Fit statistics for latent class analyses.

| Number of latent classes | Number of parameters estimated | LL | AIC | BIC |
| --- | --- | --- | --- | --- |
| 1 | 14 | -67469.87 | 134967.7 | 135076.1 |
| 2 | 29 | -65494.37 | 131046.7 | 131271.2 |
| 3 | 44 | -64801.52 | 129691 | 130031.5 |
| 4 | 59 | -64434.96 | 128987.9 | 129444.5 |
| 5 | 74 | -64275.41 | 128698.8 | 129271.5 |
| 6 | 87 | -64186.96 | 128547.9 | 129221.2 |
| 7 | 103 | -64130.76 | 128467.5 | 129364.6 |
| 8 | 118 | -64108.53 | 128453.1 | 129366.2 |
| 9 | 133 | -64050.57 | 128367.1 | 129396.4 |
| 10 | 145 | -64039.78 | 128369.6 | 129491.7 |
| 11 | 156 | -64028.26 | 128368.5 | 129575.8 |
| 12 | 176 | -63990.24 | 128332.5 | 129694.5 |
| 13 | 187 | -63998.86 | 128371.7 | 129818.9 |
| 14 | 193 | -63969.45 | 128324.9 | 129818.5 |
| ﻿LL = Log likelihood; BIC = Bayesian Information Criterion; AIC = Akaike Information Criterion | | | | |

Table B. Odds ratio from multinomial logistic regression results for categorical PM_2.5_ intensity by latent class analyses (referring to Class 4: Relatively healthy), CHARLS 2011

|  | Class 1:  Respiratory | Class 2:  Musculoskeletal | Class 3  Cardio-metabolic |
| --- | --- | --- | --- |
| PM_2.5_ intensity (ref: 0-35) | |  |  |
| *2 (36-45)* | 1.254 | 1.529*** | 1.030 |
|  | (0.882 – 1.784) | (1.239 – 1.887) | (0.768 – 1.383) |
| *3 (46-55)* | 1.944*** | 1.533*** | 1.083 |
|  | (1.405 – 2.690) | (1.225 – 1.872) | (0.817 – 1.436) |
| *4 (56-65)* | 2.030*** | 1.981*** | 1.648*** |
|  | (1.444 – 2.855) | (1.601 – 2.453) | (1.234 – 2.202) |
| *5 (66+)* | 2.527*** | 1.575*** | 2.521*** |
|  | (1.765 – 3.619) | (1.244 – 1.994) | (1.873 – 3.393) |
| 1) Covariates include age, education, HuKou-residence, occupation, marital status, smoking status, logged GDP;  2) *** p<0.001, ** p<0.01, * p<0.05, # p<0.1 | | | |

Table C. Coefficients and 95% confidence intervals of growth curve models on associations between categorical PM_2.5_ intensity and multimorbidity, CHARLS 2011-2015

|  | Model 1:  Base | Model 2: Model 1  + Education | Model 3: Model 2  + SES | Model 4: Model 3  + Smoking + GDP |
| --- | --- | --- | --- | --- |
| PM_2.5_ intensity (ref: 0-35) | |  |  |  |
| *2 (36-45)* | -0.102*** | -0.113*** | -0.118*** | -0.118*** |
|  | (-0.148 – -0.055) | (-0.160 – -0.066) | (-0.164 – -0.071) | (-0.165 – -0.072) |
| *3 (46-55)* | -0.108*** | -0.124*** | -0.135*** | -0.131*** |
|  | (-0.152 – -0.063) | (-0.169 – -0.079) | (-0.180 – -0.090) | (-0.176 – -0.086) |
| *4 (56-65)* | -0.0917*** | -0.103*** | -0.106*** | -0.106*** |
|  | (-0.135 – -0.049) | (-0.147 – -0.060) | (-0.150 – -0.063) | (-0.149 – -0.063) |
| *5 (66-75)* | -0.001 | -0.016 | -0.020 | -0.019 |
|  | (-0.046 – 0.044) | (-0.062 – 0.029) | (-0.065 – 0.026) | (-0.064 – 0.026) |
| 1) Covariates include age, age squared, education, HuKou-residence, occupation, marital status, smoking status, logged GDP;  2) *** p<0.001, ** p<0.01, * p<0.05, # p<0.1 | | | | |

Table D. Incidence rate ratio and 95% confidence intervals of growth curve models on associations of 10 $\mu g$/m^3^ increase in continuous PM_2.5_ intensity and covariates with multimorbidity among those aged 45-64, CHARLS 2011-2015

|  | Model 1:  Base | Model 2: Model 1  + Education | Model 3: Model 2  + SES | Model 4: Model 3  + Smoking + GDP |
| --- | --- | --- | --- | --- |
| PM_2.5_ | -0.220*** | -0.224*** | -0.229*** | -0.226*** |
|  | (-0.274 – -0.166) | (-0.278 – -0.171) | (-0.283 – -0.175) | (-0.279 – -0.172) |
| PM_2.5_ Square | 0.020*** | 0.020*** | 0.020*** | 0.020*** |
|  | (0.015 – 0.025) | (0.015 – 0.025) | (0.015 – 0.025) | (0.015 – 0.025) |
| Age | 0.131*** | 0.137*** | 0.139*** | 0.142*** |
|  | (0.079 – 0.184) | (0.084 – 0.189) | (0.087 – 0.192) | (0.089 – 0.194) |
| Age square | -0.0008** | -0.0008*** | -0.0008*** | -0.0009*** |
|  | (-0.001 – -0.0003) | (-0.001 – -0.0003) | (-0.001 – -0.0004) | (-0.001 – -0.0004) |
| Gender (ref: Male) |  |  |  |  |
| *Female* | 0.164*** | 0.183*** | 0.175*** | 0.207*** |
|  | (0.129 – 0.198) | (0.146 – 0.219) | (0.138 – 0.211) | (0.159 – 0.255) |
| Education (ref: no schooling) | |  |  |  |
| *Primary* |  | 0.101*** | 0.096*** | 0.101*** |
|  |  | (0.053 – 0.149) | (0.048 – 0.144) | (0.052 – 0.149) |
| *Middle +* |  | 0.072** | 0.044 | 0.051# |
|  |  | (0.022 – 0.123) | (-0.011 – 0.098) | (-0.004 – 0.105) |
| HuKou (ref: Rural) |  |  |  |  |
| *Rural-urban* |  |  | -0.031 | -0.021 |
|  |  |  | (-0.076 – 0.014) | (-0.066 – 0.024) |
| *Urban* |  |  | 0.081** | 0.088*** |
|  |  |  | (0.032 – 0.129) | (0.040 – 0.137) |
| Occupation (ref: agricultural) | |  |  |  |
| *Non-agricultural* |  |  | 0.002 | 0.003 |
|  |  |  | (-0.041 – 0.045) | (-0.040 – 0.046) |
| *Managerial* |  |  | -0.043 | -0.044 |
|  |  |  | (-0.101 – 0.015) | (-0.102 – 0.014) |
| Marital (ref: partnered) | |  |  |  |
| *Single* |  |  | 0.062* | 0.064* |
|  |  |  | (0.004 – 0.120) | (0.007 – 0.122) |
| Smoking (ref: Never) |  |  |  |  |
| *Former* |  |  |  | 0.191*** |
|  |  |  |  | (0.138 – 0.244) |
| *Current* |  |  |  | 0.004 |
|  |  |  |  | (-0.042 – 0.051) |
| Log GDP |  |  |  | -0.038** |
|  |  |  |  | (-0.066 – -0.010) |
| Constant | -4.328*** | -4.559*** | -4.585*** | -4.294*** |
|  | (-5.774 – -2.882) | (-6.010 – -3.108) | (-6.036 – -3.134) | (-5.760 – -2.829) |
| **Random effects** |  |  |  |  |
| Variance |  |  |  |  |
| *Individuals (age)* | 7.09e-15*** | 6.82e-15*** | 6.20e-15*** | 1.04e-14*** |
| *Years* | 0.659*** | 0.658*** | 0.654*** | 0.646*** |
| Covariance |  |  |  |  |
| *Individuals – Years* | 6.53e-11 | 6.34e-11 | 5.87e-11 | 9.59e-11 |
|  |  |  |  |  |
| Log likelihood | -46723.281 | -46714.865 | -46702.602 | -46663.209 |
|  |  |  |  |  |
| Observations | 31,780 | 31,780 | 31,780 | 31,780 |
| Number of IDs | 14,207 | 14,207 | 14,207 | 14,207 |
| *** p<0.001, ** p<0.01, * p<0.05, # p<0.1 | | | | |

Table E. Incidence rate ratio and 95% confidence intervals of growth curve models on associations of 10 $\mu g$/m^3^ increase in continuous PM2.5 intensity and covariates with multimorbidity among those aged 65-85, CHARLS 2011-2015

|  | Model 1:  Base | Model 2: Model 1  + Education | Model 3: Model 2  + SES | Model 4: Model 3  + Smoking + GDP |
| --- | --- | --- | --- | --- |
| PM_2.5_ | -0.113** | -0.122*** | -0.130*** | -0.127*** |
|  | (-0.181 – -0.045) | (-0.190 – -0.054) | (-0.198 – -0.063) | (-0.194 – -0.060) |
| PM_2.5_ Square | 0.0140*** | 0.014*** | 0.015*** | 0.015*** |
|  | (0.008 – 0.020) | (0.008 – 0.020) | (0.009 – 0.021) | (0.009 – 0.021) |
| Age | 0.104* | 0.101* | 0.097* | 0.093* |
|  | (0.019 – 0.189) | (0.016 – 0.186) | (0.0120 – 0.182) | (0.009 – 0.177) |
| Age square | -0.0007* | -0.0006* | -0.0006* | -0.0006# |
|  | (-0.001 – -0.0006) | (-0.001 – -0.0002) | (-0.001 – -0.0007) | (-0.001 – 0.0001) |
| Gender (ref: Male) |  |  |  |  |
| *Female* | 0.108*** | 0.170*** | 0.164*** | 0.171*** |
|  | (0.0671 – 0.149) | (0.126 – 0.213) | (0.120 – 0.209) | (0.118 – 0.224) |
| Education (ref: no schooling) | |  |  |  |
| *Primary* |  | 0.174*** | 0.153*** | 0.153*** |
|  |  | (0.125 – 0.223) | (0.104 – 0.203) | (0.103 – 0.203) |
| *Middle +* |  | 0.200*** | 0.121*** | 0.118*** |
|  |  | (0.139 – 0.261) | (0.052 – 0.190) | (0.050 – 0.187) |
| HuKou (ref: Rural) |  |  |  |  |
| *Rural-urban* |  |  | -0.035 | -0.032 |
|  |  |  | (-0.091 – 0.022) | (-0.089 – 0.025) |
| *Urban* |  |  | 0.139*** | 0.136*** |
|  |  |  | (0.083 – 0.195) | (0.079 – 0.192) |
| Occupation (ref: agricultural) | |  |  |  |
| *Non-agricultural* |  |  | -0.011 | -0.018 |
|  |  |  | (-0.063 – 0.042) | (-0.070 – 0.033) |
| *Managerial* |  |  | -0.0320 | -0.0317 |
|  |  |  | (-0.179 – 0.115) | (-0.179 – 0.116) |
| Marital (ref: partnered) | |  |  |  |
| *Single* |  |  | -0.013 | -0.010 |
|  |  |  | (-0.058 – 0.033) | (-0.056 – 0.035) |
| Smoking (ref: Never) |  |  |  |  |
| *Former* |  |  |  | 0.170*** |
|  |  |  |  | (0.116 – 0.224) |
| *Current* |  |  |  | -0.064* |
|  |  |  |  | (-0.115 – -0.012) |
| Log GDP |  |  |  | -0.007 |
|  |  |  |  | (-0.039 – 0.025) |
| Constant | -3.109 | -4.312 | -3.407 | -3.564 |
|  | (-10.19 – 3.976) | (-11.37 – 2.748) | (-10.46 – 3.640) | (-10.55 – 3.425) |
| **Random effects** |  |  |  |  |
| Variance |  |  |  |  |
| *Individuals (age)* | 2.45e-05*** | 1.78e-05*** | 1.59e-05*** | 1.15e-05*** |
| *Years* | 0.088*** | 0.119*** | 0.128*** | 0.153*** |
| Covariance |  |  |  |  |
| *Individuals – Years* | 0.00147*** | 0.0015*** | 0.0014*** | 0.0013*** |
|  |  |  |  |  |
| Log likelihood | -23773.023 | -23743.529 | -23727.006 | -23680.922 |
|  |  |  |  |  |
| Observations | 14,008 | 14,008 | 14,008 | 14,008 |
| Number of IDs | 6,758 | 6,758 | 6,758 | 6,758 |
| *** p<0.001, ** p<0.01, * p<0.05, # p<0.1 | | | | |

Table F. Coefficients and 95% confidence intervals of the associations of PM_2.5_ exposure and covariates with multimorbidity using complete case and imputed case samples, CHARLS 2011-2015

|  | Complete case samples | | Imputed case samples | |
| --- | --- | --- | --- | --- |
|  | Model 1 | Model 2 | Model 1 | Model 2 |
| PM_2.5_ intensity | -0.206*** | -0.202*** | -0.171^***^ | -0.167^***^ |
|  | (-0.251 – -0.162) | (-0.246 – -0.158) | (-0.209 – -0.132) | (-0.205 – -0.129) |
| PM_2.5_ intensity square | 0.019*** | 0.0191*** | 0.016^***^ | 0.016^***^ |
|  | (0.015 – 0.023) | (0.0151 – 0.0231) | (0.013 – 0.020) | (0.012 – 0.019) |
| Age | 0.136*** | 0.137*** | 0.091^***^ | 0.092^***^ |
|  | (0.121 – 0.152) | (0.122 – 0.153) | (0.077 – 0.105) | (0.078 – 0.105) |
| Age square | -0.0009*** | -0.0009*** | -0.0005^***^ | -0.0005^***^ |
|  | (-0.001 – -0.0007) | (-0.001 – -0.0007) | (-0.0006 – -0.0004) | (-0.0007 – -0.0004) |
| Gender (ref: Male) | |  |  |  |
| *Female* | 0.181*** | 0.197*** | 0.161^***^ | 0.180^***^ |
|  | (0.151 – 0.211) | (0.159 – 0.235) | (0.134 – 0.187) | (0.146 – 0.214) |
| Education (ref: no schooling) | |  |  |  |
| *Primary* | 0.135*** | 0.135*** | 0.124^***^ | 0.124^***^ |
|  | (0.0977 – 0.172) | (0.0980 – 0.173) | (0.091 – 0.157) | (0.091 – 0.157) |
| *Middle +* | 0.0860*** | 0.0869*** | 0.060^**^ | 0.062^**^ |
|  | (0.0416 – 0.130) | (0.0425 – 0.131) | (0.022 – 0.098) | (0.0232 – 0.100) |
| HuKou (ref: Rural) |  |  |  |  |
| *Rural-urban* | -0.0286 | -0.0250 | -0.020 | -0.015 |
|  | (-0.0654 – 0.00828) | (-0.0623 – 0.0123) | (-0.053 – 0.014) | (-0.049 – 0.019) |
| *Urban* | 0.105*** | 0.107*** | 0.118^***^ | 0.121^***^ |
|  | (0.066 – 0.144) | (0.068 – 0.146) | (0.084 – 0.153) | (0.086 – 0.156) |
| Occupation (ref: agricultural) | |  |  |  |
| *Non- agricultural* | -0.008 | -0.0121 | 0.002 | -0.001 |
|  | (-0.043 – 0.026) | (-0.0463 – 0.0221) | (-0.028 – 0.033) | (-0.031 – 0.029) |
| *Managerial* | -0.042 | -0.044 | -0.058^*^ | -0.059^*^ |
|  | (-0.094 – 0.011) | (-0.097 – 0.009) | (-0.108 – -0.007) | (-0.109 – -0.008) |
| Marital (ref: partnered) | |  |  |  |
| *Single* |  | 0.017 |  | 0.015 |
|  |  | (-0.021 – 0.054) |  | (-0.020 – 0.049) |
| Smoking (ref: Never) |  |  |  |  |
| *Former* |  | 0.175*** |  | 0.192^***^ |
|  |  | (0.137 – 0.214) |  | (0.154 – 0.229) |
| *Current* |  | -0.024 |  | -0.024 |
|  |  | (-0.060 – 0.012) |  | (-0.059 – 0.012) |
| Log GDP |  | -0.014 |  | -0.018 |
|  |  | (-0.036 – 0.008) |  | (-0.038 – 0.002) |
| Constant | -4.507*** | -4.385*** | -2.973^***^ | -2.815^***^ |
|  | (-5.008 – -4.005) | (-4.931 – -3.838) | (-3.416 – -2.530) | (-3.287 – -2.342) |
| **Random effects** |  |  |  |  |
| Variance (*Individuals*) | 0.602*** | 0.593*** | 0.473^***^ | 0.465^***^ |
|  | (0.580 – 0.625) | (0.571 – 0.615) | (0.455 – 0.491) | (0.448 – 0.482) |
|  |  |  |  |  |
| Log likelihood | -69825.48 | -69751.124 |  |  |
|  |  |  |  |  |
| Observations | 45,788 | 45,788 | 53,357 | 53,357 |
| Number of IDs | 19,098 | 19,098 | 23,125 | 23,125 |
| *** p<0.001, ** p<0.01, * p<0.05, # p<0.1 | | | | |

﻿**Fig A**. Relative fit for latent class analyses (BIC, AIC).
